# Lipid profiling reveals *Leymus Chinensis* root insensitivity to Ca limitation

**DOI:** 10.1186/s12870-023-04627-8

**Published:** 2023-11-30

**Authors:** Yang Nan, Yanbing Dong, Lili Zhang, Lijuan Zhang, Zhi Qi, Haiye Luan, Ju Yang

**Affiliations:** 1 Key Laboratory of Forage and Endemic Crop Biology, Ministry of Education, School of Life Sciences, Inner Mongolia University, Hohhot, China; 2 College of Ocean and Bioengineering, Yancheng Teachers University, Yancheng, China

**Keywords:** *Leymus Chinensis*, *Arabidopsis*, Root, Lipid composition, Endocytosis, Calcium limitation

## Abstract

**Background:**

*Leymus chinensis* (*L. chinensis*) is a perennial native forage grass widely distributed in the steppe of Inner Mongolia as the dominant species. Calcium (Ca) is an essential mineral element important for plant adaptation to the growth environment. Ca limitation was previously shown to strongly inhibit *Arabidopsis (Arabidopsis thaliana)* seedling growth and disrupt plasma membrane stability and selectivity, increasing fluid-phase-based endocytosis and contents of all major membrane lipids.

**Results:**

In this study, we investigated the significance of Ca for *L. chinensis* growth and membrane stability relative to *Arabidopsis*. Our results showed that Ca limitation did not affect *L. chinensis* seedling growth and endocytosis in roots. Moreover, the plasma membrane maintained high selectivity. The lipid phosphatidylcholine (PC): phosphatidylethanolamine (PE) ratio, an indicator of the membrane stability, was five times higher in *L. chinensis* than in *Arabidopsis*. Furthermore, in *L. chinensis*, Ca limitation did not affect the content of any major lipid types, decreased malondialdehyde (MDA) content, and increased superoxide dismutase (SOD) activity, showing an opposite pattern to that in *Arabidopsis*. *L. chinensis* roots accumulated higher contents of PC, phosphatidylinositol (PI), monogalactosyldiacylglycerol (MGDG), phosphatidylglycerol (PG), cardiolipin (CL), digalactosyldiacylglycerol (DGDG), and lysophosphatidylcholine (LPC) but less phosphatidylethanolamine (PE), diacylglycerol (DAG), triacylglycerolv (TAG), phosphatidylserine (PS), lysobisphosphatidic acids (LPAs), lysophosphatidylethanolamine (LPE), and lysophosphatidylserine (LPS) than *Arabidopsis* roots. Moreover, we detected 31 and 66 unique lipids in *L. chinensis* and *Arabidopsis*, respectively.

**Conclusions:**

This study revealed that *L. chinensis* roots have unique membrane lipid composition that was not sensitive to Ca limitation, which might contribute to the wider natural distribution of this species.

**Supplementary Information:**

The online version contains supplementary material available at 10.1186/s12870-023-04627-8.

## Background

*Leymus chinensis* (Trin.) Tzvel is a perennial gramineous Triticeae grass belonging to the genus *Leymus* that serves as an highly nutritious forage for grazing livestock [1, 2]. It is widely distributed in the eastern Eurasian steppe as the dominant species for the ecological conservation of arid and semiarid regions [3, 4]. How this grass adapts to such diverse growth environments is largely unknown.

In plant cells, calcium (Ca) is an essential macronutrient that plays vital roles in helping plant cells decipher interior and exterior cues to make optimal responses [5]. In both plant and animal cells, Ca^2+^ concentrations in the cytosol and within organelles are regulated by sophisticated mechanisms linked with almost every aspect of cellular signaling [6]. Ca in all living organisms is ultimately derived from the soil and water bodies on the earth surface. Unfortunately, several long-term ecological studies have uncovered a significant declining trend of Ca content in forest soils and lakes [7, 8]. Ca limitation has adverse effects not only on plant growth and development, but also on plant responses to biotic and abiotic stresses [9, 10]. How plant cells cope with low Ca availability is not fully understood.

In animal cells, the drop in extracellular Ca^2+^ concentrations is monitored by the calcium-sensing receptor (CaSR). It regulates endocytosis and exocytosis to maintain cell membrane integrity and intercellular communication [11, 12]. In *Arabidopsis*, Ca limitation decreases membrane stability and selectivity [13].

Ca^2+^ interacts with lipids in the cell bilayer membrane to regulate membrane homeostasis [14]. Amphipathic glycerolipids are the dominant type of lipids in most cells. They have a glycerol backbone with nonpolar fatty acids on their *sn*-1 and *sn*-2 position and a polar headgroup on *sn*-3. The major fatty acids of plants are synthesized in the plastid and have a chain length of 16 or 18 carbons with one to three *cis* double bonds [15].

Glycerolipid biosynthesis takes place in the plastid inner envelope and the endoplasmic reticulum (ER). In both locations, the first step leads to the formation of phosphatidic acid (PA). In plastids, PA can be directly converted to phosphatidylglycerol (PG) or produce monogalactosyldiacylglycerol (MGDG) and digalactosyldiacylglycerol (DGDG) through diacylglycerol (DAG) [15]. In the ER, PA can be used to synthesize the phospholipids phosphatidylinositol (PI), phosphatidylserine (PS), PG, phosphatidylethanolamine (PE), and phosphatidylcholine (PC). PG can be converted to cardiolipin (CL) in the mitochondrion inner envelope. When PA, PC, PE, or PS lacks one fatty acid chain at their *sn*-1 or *sn*-2 position, they yield lysobisphosphatidic acids (LPAs), lysophosphatidylcholine (LPC), lysophosphatidylethanolamine (LPE), and lysophosphatidylserine (LPS) [16]. PC and PE exist in the outer and inner leaflet of the bilayer membrane. A higher PC to PE ratio tends to be associated with more stable membrane [17].

Polar glycerolipids are primarily classified according to the structure of the polar head group. Each polar glycerolipid class is subclassified according to the fatty acid species in the hydrophobic tails, which vary in the number of carbon chains and the number and position of unsaturated bonds [18]. With advances in lipid profiling techniques, namely, “lipidomics”, hundreds of lipids have been identified in plants [19]. Modifying membrane lipid composition is recognized as an efficient and universal strategy that plants utilize to cope with various growth environments.

Significant decreasing of the Ca contents in surface soils has been reported in different ecological systems worldwide [20–22]. It was reported previously that the low nitrogen availability in the northern China grassland limits *L. chinensis* growth [23]. *L. chinensis* habitat region is characterized as calcareous soil and there is no data available for the changing pattern of the calcium contents in the surface soil. However, *L. chinensis* could be confronted with physiological calcium limitation caused by widely existing drought and salinity in the arid and semiarid region [24, 25].

Besides of calcium, under various environmental stress condition, the reactive oxygen species (ROS) negatively affect membrane integrity by inducing the membrane lipid peroxidation, which could disturb the lipid bilayer order and membrane structure [26]. ROS themselves have profound regulative effects on intracellular calcium distribution [27]. Calcium, ROS and lipids could be linked by annexins with both calcium and lipid binding capacity [28].

In this study, we discovered that Ca limitation had surprisingly no obvious inhibitory effect on the vegetative growth of *L. chinensis* young seedlings. Moreover, we hypothesize that the unique membrane lipid composition of *L. chinensis* is a key factor in its wide distribution in the eastern Eurasian steppe, compared with the model plant *Arabidopsis* (*Arabidopsis thaliana*).

## Results

### Effect of Ca limitation on *L. chinensis* growth and Ca content

**Fig. 1:**
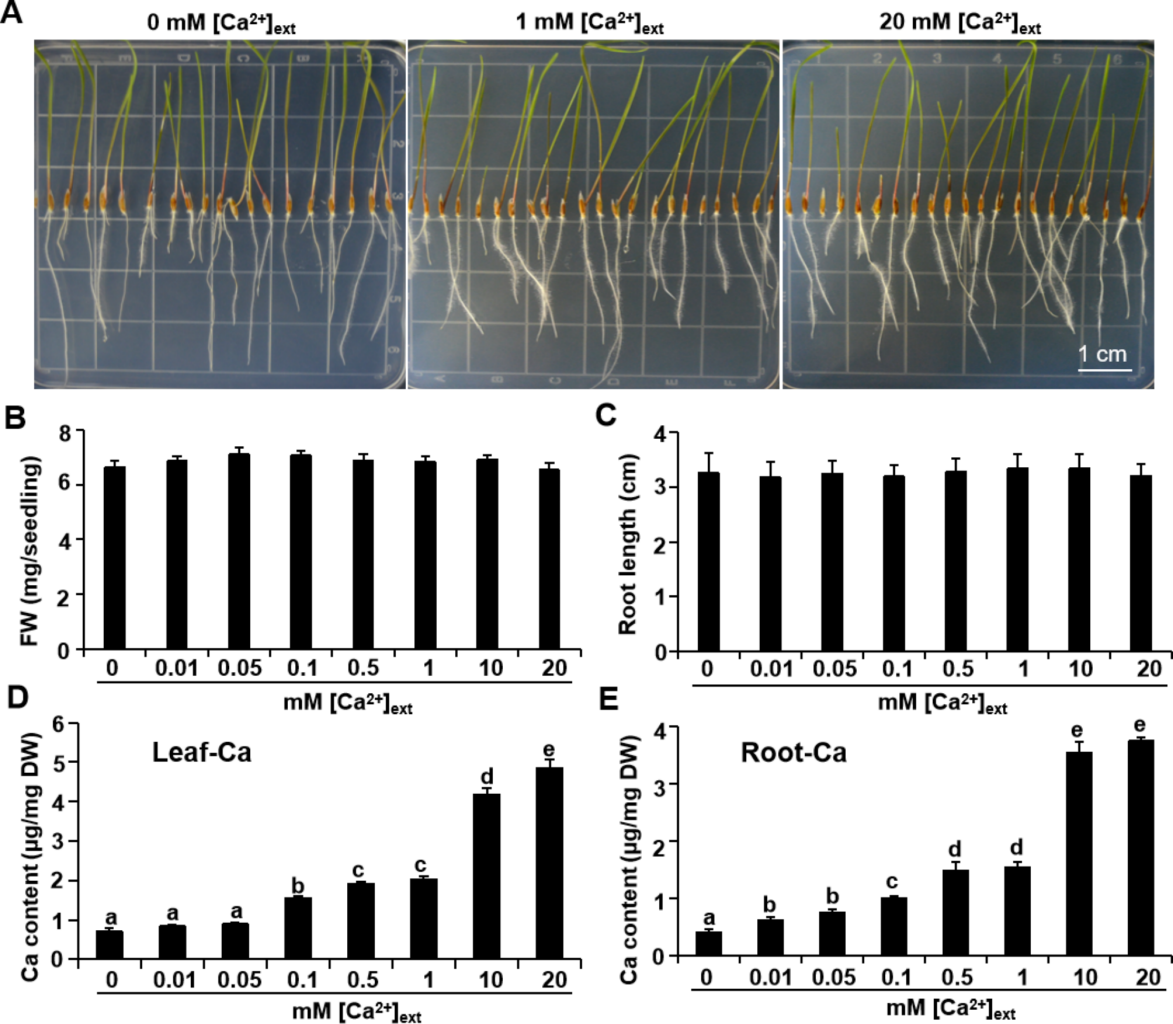
Effects of different [Ca^2+^]_ext_ on the growth and Ca content of *L. chinensis*. *L. chinensis* seeds were sown on medium with various Ca^2+^ concentrations, incubated in the dark at 30 °C for 2 d, before being grown vertically in a growth chamber for 7 d. **(A)** Typical growth phenotype of *L. chinensis* on growth media with different [Ca^2+^]_ext_ (0, 1, or 20 mM Ca^2+^); scale bar, 1 cm. Effect of [Ca^2+^]_ext_ on seedling fresh weight (FW) **(B)** and root length **(C)**. Data represents average of 20 seedlings from one of three typical biological experiments. **(D)** Leaf Ca content and **(E)** root Ca content. Data represents measurements from six groups of seedlings with 10 seedlings per group from one of three typical biological experiments. Different lowercase letters indicate significant difference among treatments. One-way ANOVA followed by Tukey multiple comparisons test, *p* < 0.05. DW, dry weight; FW, fresh weight

We grew *L. chinensis* seedlings vertically on solid medium supplied with different concentrations of Ca^2+^ (designated as [Ca^2+^]_ext_ for exogenously supplied Ca^2+^) for seven days. At the end of this growth period, both leaves and roots reached the edge of the Petri dish (Fig. 1A). Ca limitation (0 mM of Ca^2+^ added to the medium) had no inhibitory effects on seedling fresh weight (Fig. 1B) or root elongation (Fig. 1C). We conclude that *L. chinensis* seedling growth shows no dependence on [Ca^2+^]_ext_ present in the medium over a 0–20 mM range (Fig. 1B and C).

By contrast, the *L. chinensis* seedling growth was strongly and significantly inhibited by the absence of nitrogen (N) in the growth medium (Fig. S1), which demonstrates that seedlings were mature enough under our growth conditions to take up mineral nutrients from their surrounding environment. Likewise, the Ca content in both leaves and roots should decline with decreasing [Ca^2+^]_ext_ in the growth medium. Indeed, the Ca content in the leaves (Fig. 1D) and roots (Fig. 1E) of seedlings grown on the 0 mM [Ca^2+^]_ext_ condition reached about 10% of the Ca content in those grown in the presence of 20 mM [Ca^2+^]_ext_. This result suggests that *L. chinensis* seedlings are tolerant to Ca limitation.

### *L. chinensis* oxidative responses to Ca limitation

**Fig. 2:**
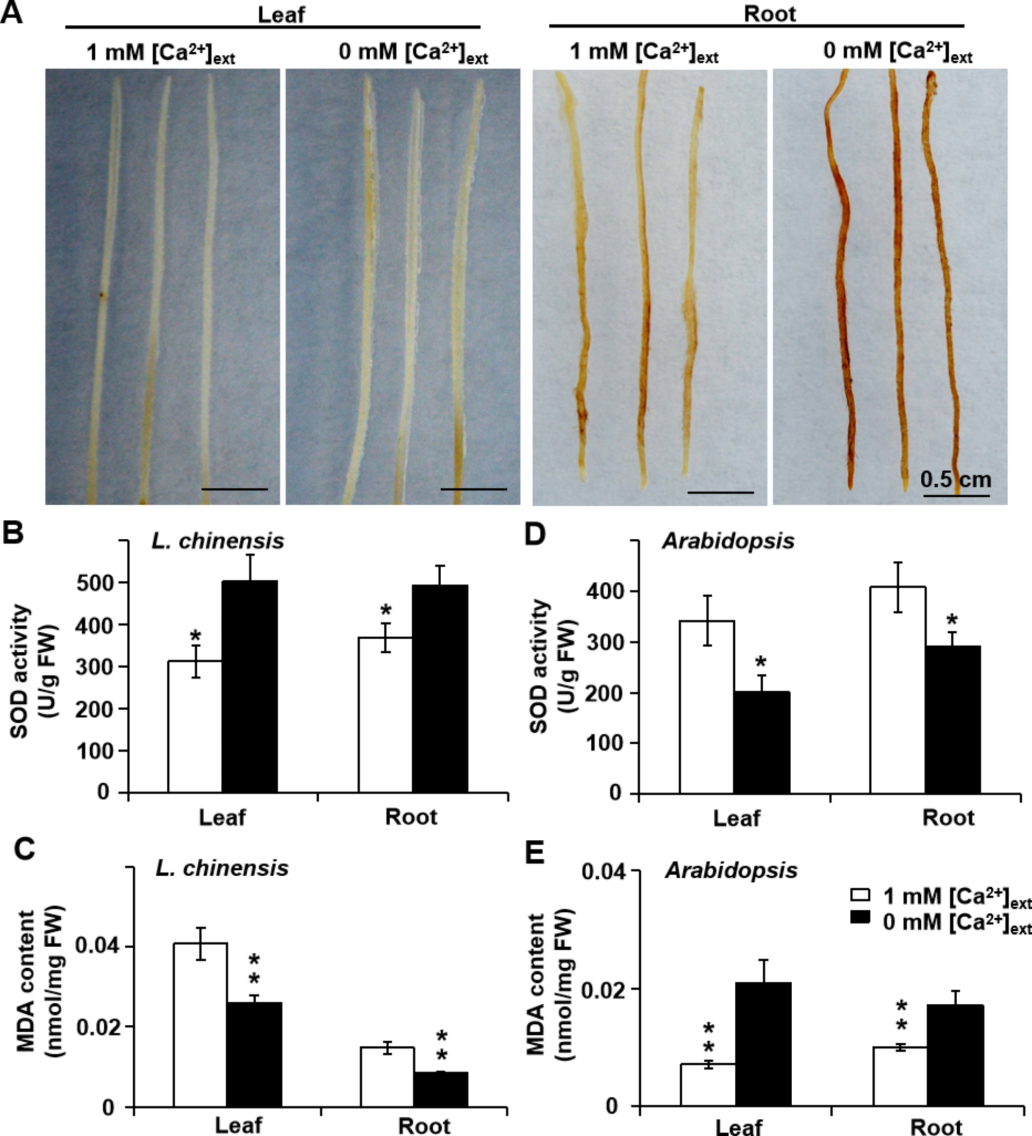
* L. chinensis* oxidative responses to the Ca limitation. Plant materials were grown as in Fig. 1. **(A)** Typical *L. chinensis* leaves (left) and roots (right) stained with DAB for H_2_O_2_ localization under control conditions (1 mM [Ca^2+^]_ext_) or 0 mM [Ca^2+^]_ext_. **(B)** SOD activity and **(C)** MDA content in *L. chinensis* leaves and roots. **(D)** SOD activity and **(E)** MDA content in *Arabidopsis* leaves and roots. *n* = 5. *p < 0.05, **p < 0.01 with Student’s *t*-test

H_2_O_2_ accumulation is a common plant oxidative response to environmental stresses (26,27), which prompted us to test the locations and levels of reactive oxygen species in *L. chinensis* seedlings. We determined that Ca limitation induces strong H_2_O_2_ production in the roots but not the leaves of *L. chinensis* seedlings, compared with seedlings grown in the presence of 1 mM [Ca^2+^]_ext_, as indicated by the formation of a brown precipitate following incubation with 3,3′-diaminobenzidine (DAB) (Fig. 2A).

Ca limitation raised the superoxide dismutase (SOD) activity relative to the 1 mM [Ca^2+^]_ext_ condition (Fig. 2B) and lowered MDA content in *L. chinensis* leaves and roots (Fig. 2C). On the contrary, it decreased SOD activity (Fig. 2D) and increased malondialdehyde (MDA) content in *Arabidopsis* leaves and roots (Fig. 2E). Meanwhile, Ca limitation had no significant effect on catalase (CAT) and peroxidase (POD) activities in *L. chinensis* leaves and roots (Fig. S2A, S2C), but suppressed the two in *Arabidopsis* leaves and root (Fig. S2B, S2D).

### Effect of Ca limitation on *L. chinensis* root endocytosis

**Fig. 3:**
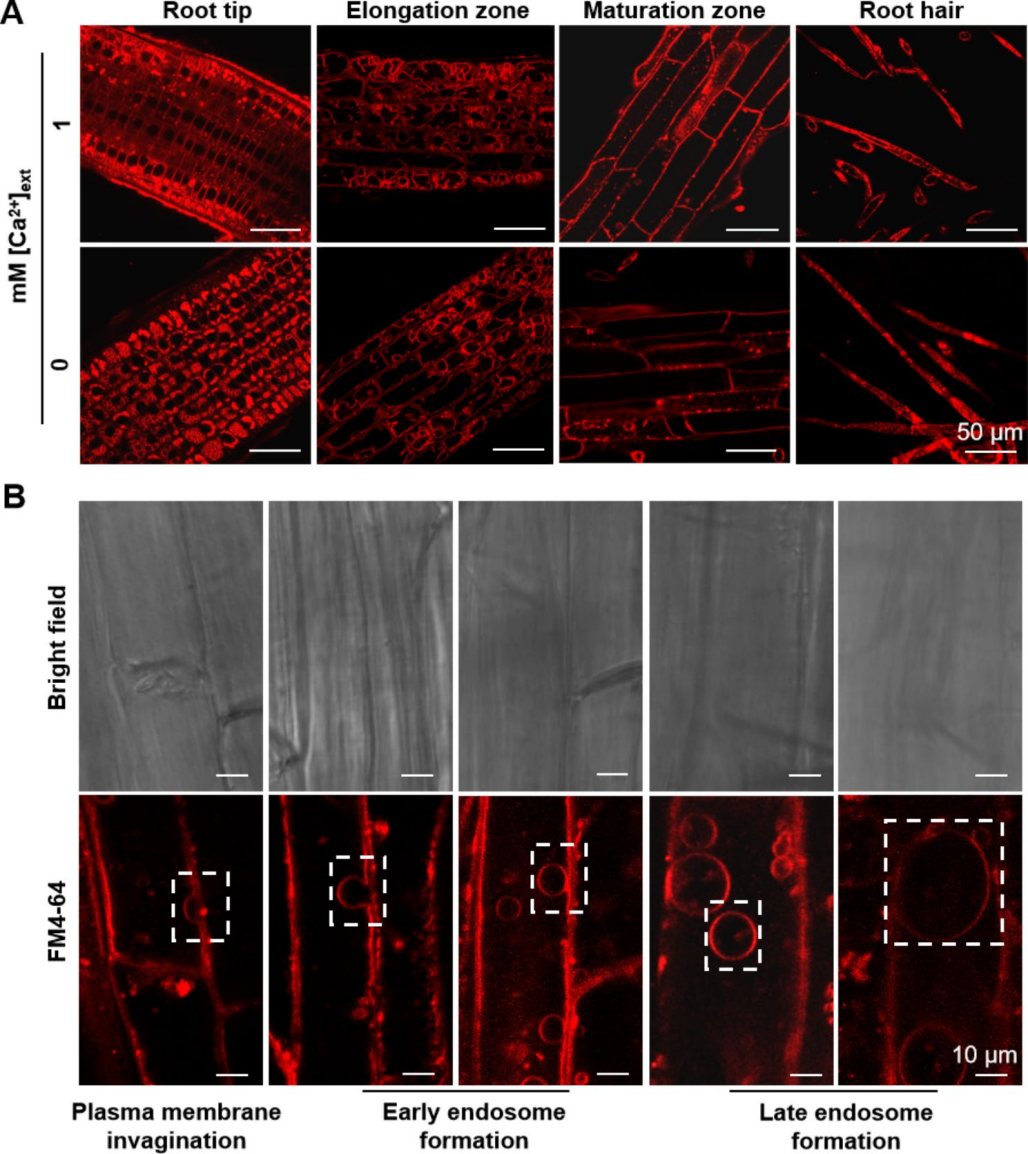
Endocytosis in different regions of *L. chinensis* roots and intracellular stages of endocytosis. Plant materials were grown as in Fig. 1. The endomembrane system was monitored by incubating the roots with the fluorescent membrane probe FM4-64 for 30 min before imaging. Typical bright-field and confocal scanning laser microscopy images are shown. **(A)** Endocytosis in different regions of *L. chinensis* roots under control conditions (1 mM [Ca^2+^]_ext_) or 0 mM [Ca^2+^]_ext_ treatment. **(B)** Intracellular stages of endocytosis in *L. chinensis* roots. The dotted rectangles highlight typical endocytosis steps

We examined *L. chinensis* seedlings grown on medium containing either 1 mM (control, CK) or no [Ca^2+^]_ext_ for endocytosis activity in their root cells, using the fluorescence membrane probe FM4-64. We observed extensive endocytosis activity in cells at the root tip, elongation zones, maturation zones, and root hairs (Fig. 3A). Notably, we observed no difference between the control and Ca limitation conditions (Fig. 3A).

We chose the cells in the maturation zone to characterize endocytosis in detail. We detected all conserved steps of endocytosis in these cells: initial plasma membrane invagination, followed by early and late endosome formation, which showed no obvious difference between the seedlings grown on the CK and [Ca^2+^]_ext_ limitation media (Fig. 3B).

### Effect of Ca limitation on *L. chinensis* root membrane selectivity

**Fig. 4:**
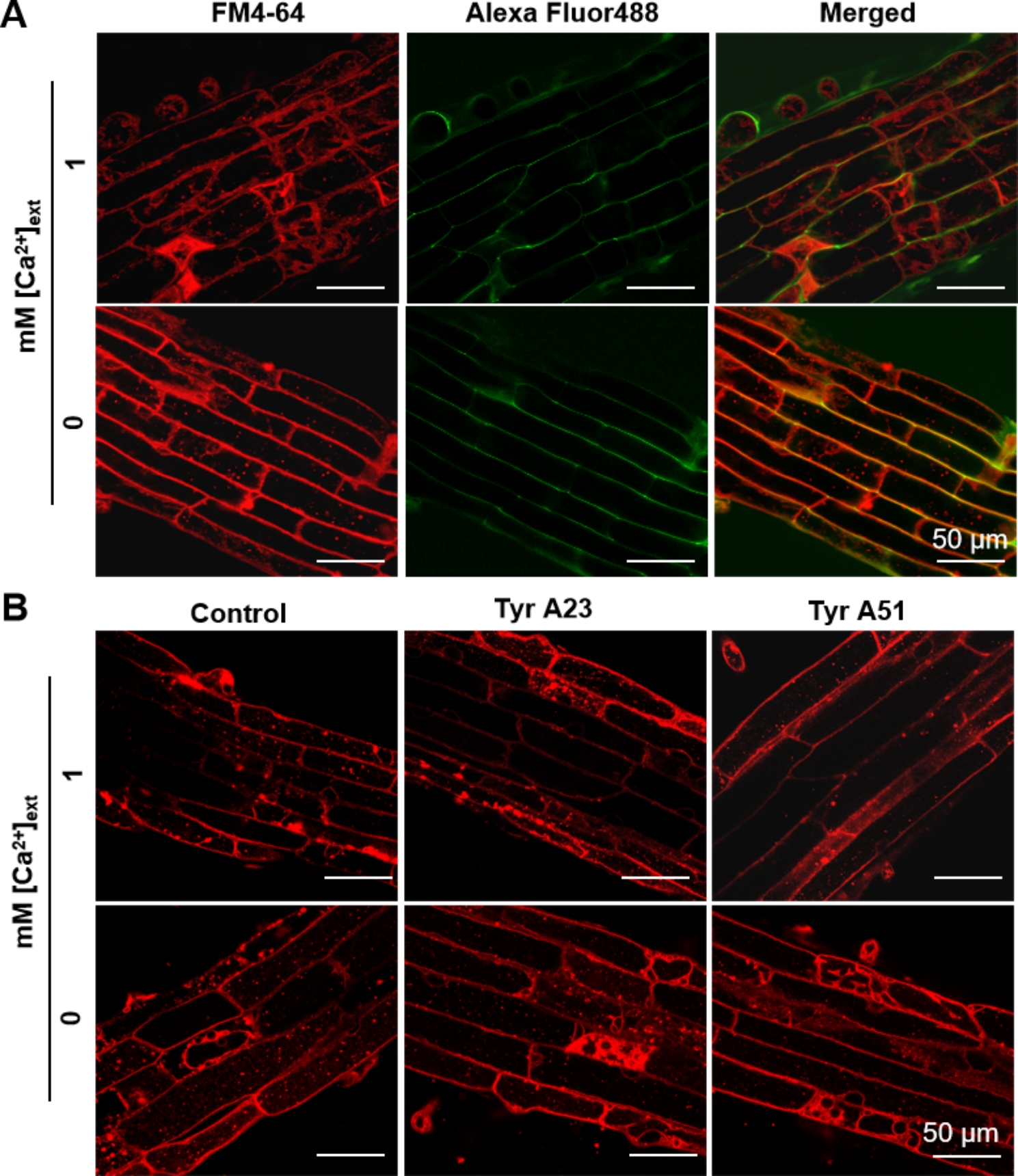
Effect of Ca limitation on *L. chinensis* root membrane selectivity. Plant materials were grown as in Fig. 1; the imaging procedure was as in Fig. 3. **(A)** Alexa Fluor 488, a membrane-impermeable green fluorescent dye without biological activity, was used to track specific fluid-phase-based endocytosis. *L. chinensis* seedlings were co-stained with FM4-64 and Alexa Fluor 488 for 30 min. **(B)** Tyr A23, an inhibitor of receptor-mediated endocytosis, and Tyr A51, a structural analog of Tyr A23 without inhibitory activity, served as the control. *L. chinensis* seedlings were treated with 30 µM Tyr A23 or Tyr A51 in corresponding solution for 1 h and then dyed for 30 min. Scale bars, 50 μm

Alexa Fluor 488 is a membrane-impermeable green fluorescent dye without biological activity and serves as a convenient probe to track nonspecific fluid-phase endocytosis activity and an indicator of membrane selectivity [29]. Accordingly, we stained the roots of *L. chinensis* seedlings grown in control condition (1 mM [Ca^2+^]_ext_) and detected green fluorescence exclusively distributed around the cell boundary, with no signals inside the cells. This indicates that the cell plasma membrane remains highly selective, preventing the Alexa Fluor 488 dye from entering the cells through nonselective fluid-phase-based endocytosis (Fig. 4A). The Ca limitation condition (0 mM) had no obvious effects on the fluorescence distribution pattern, which suggests that membrane selectivity is not sensitive to the [Ca^2+^]_ext_ used (Fig. 4A).

Moreover, the endocytosis activity in roots grown on either [Ca^2+^]_ext_ was insensitive to treatment with Tyr A23, an inhibitor of receptor-mediated endocytosis, or Tyr A51, a structural analog of Tyr A23 without inhibitory activity, which served as the control (Fig. 4B).

### Effect of Ca limitation on the profile of *L. chinensis* root lipids

**Fig. 5:**
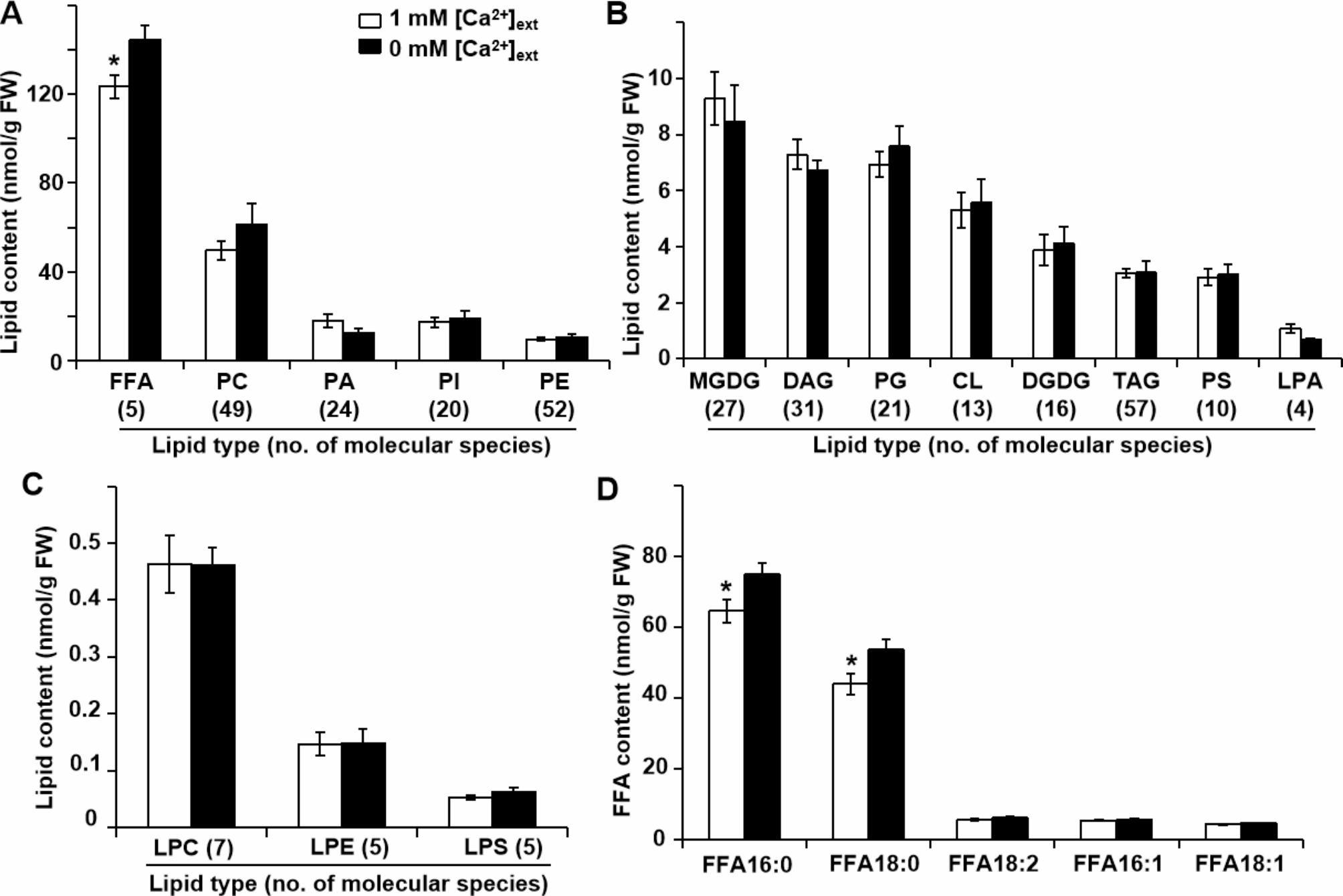
Effect of Ca limitation on the lipid profiles of *L. chinensis* roots. Plant materials were grown as in Fig. 1. Lipids were grouped into three distinct categories in terms of abundance: those greater than 10 nmol/g FW **(A)**; greater than 1 but less than 10 nmol/g FW **(B)**, and less than 1 nmol/g FW **(C)**. The number of lipid molecular species in each type is shown in brackets. **(D)** Contents of the five molecular species of free fatty acids (FFAs). Values are means ± standard errors of five biological replicates. *p < 0.05 with Student’s *t*-test. FW, fresh weight

We determined the lipid profiles of *L. chinensis* roots growth on various [Ca^2+^]_ext_ and thus detected 16 types of membrane and storage lipids. Free fatty acid (FFA), PC, PA, PI, and PE were the dominant types of lipids, with content over 10 nmol/g fresh weight (FW). Ca limitation induced a significant increase in FFA content in the roots (Fig. 5A) but had no significant effects on the other 15 types of lipids (Fig. 5A and B C).

The 16 types of lipids corresponded to 347 individual lipid molecular species (Tables S1, S2). FFA lipids consisted of five molecular species: FFA 16:0, 16:1; 18:0, 18:1, and 18:2. We noticed a significant increase in the content of the two dominant species FFA 16:0 and 18:0 in response to Ca limitation (Fig. 5D).

PC was the most abundant membrane lipid in *L. chinensis* roots and included a group of 49 molecular species with various carbon chain lengths, as well as various numbers and positions of double bonds (Fig. 5A). Two molecular species (PC 43:2, 44:3) significantly increased, while another two PC species (32:2, 32:1) decreased in the roots of *L. chinensis* grown under Ca limitation compared with the control (Table S2). The four PC species above accounted for 0.77 nmol/g FW or about 1.5% of the total PC (50 nmol/g FW), resulting in no significant change in the total PC content under Ca limitation. Similarly, another 37 lipid molecular species from 14 types of lipids showed significantly changing content in response to Ca limitation (Table S2).

### Lipid composition in *L. chinensis* and *Arabidopsis* roots

**Fig. 6:**
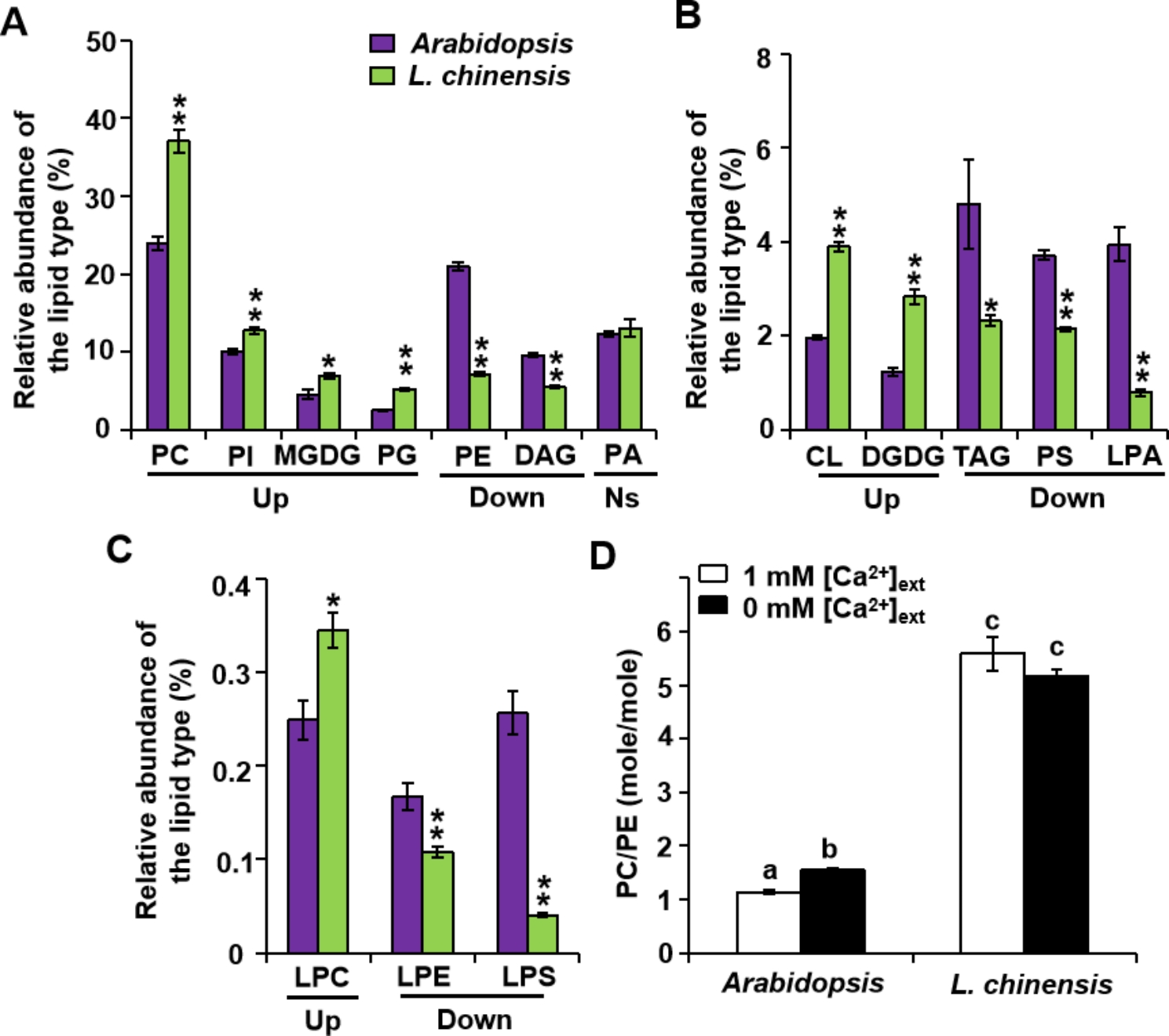
Lipid composition in the roots of *L. chinensis* and *Arabidopsis*. Abundance of each lipid type relative to the total lipids from the roots of *L. chinensis* and *Arabidopsis* (**A, B, C**). “Up” and “Down” refer to lipids existing in higher or lower amounts in *L. chinensis* than in *Arabidopsis*, respectively; Student’s *t*-test, *p* < 0.05; *n* = 5. *p < 0.05, **p < 0.01 with Student’s *t*-test. Ns, no significant difference. **(D)** Molar ratio of PC to PE in the roots of *L. chinensis* and *Arabidopsis* grown under control conditions (1 mM [Ca^2+^]_ext_) or 0 mM [Ca^2+^]_ext_ conditions. Different lowercase letters indicate significant difference among treatments. Two-way ANOVAs with Tukey post hoc multiple comparison test, *p* < 0.05

We grew *L. chinensis* and *Arabidopsis* seedlings under the same conditions of Ca limitation (0 mM [Ca^2+^]_ext_) and control conditions (1 mM [Ca^2+^]_ext_) and extracted lipids from their roots. We observed that the abundance of PC, PI, MGDG, PG, CL, DGDG, and LPC relative to the total lipid in *L. chinensis* roots is significantly higher than that in *Arabidopsis* (Fig. 6). The content of PE, DAG, TAG, PS, LPA, LPE, and LPS in *L. chinensis* was significantly lower than that in *Arabidopsis* (Fig. 6A, B and C). The lipid type with the greatest difference in relative abundance between the roots of the two plant species was LPS, representing 0.04% in *L. chinensis* and 0.26% in *Arabidopsis* or a 6.5-fold difference (Fig. 6C).

The mole ratio of PC to PE in *Arabidopsis* roots dramatically increased as a result of Ca^2+^ limitation [13], but it remained constant and high in *L. chinensis* roots (Fig. 6D). In addition, the mole ratio in *L. chinensis* roots was about five times higher than that in *Arabidopsis* (Fig. 6D). By comparing the lipid profiles in the roots of *L. chinensis* and *Arabidopsis*, we identified 31 lipid molecular species specific to *L. chinensis* (Table S3) and 66 specific to *Arabidopsis* (Table S4).

## Discussion

Ca is an essential mineral element for plant growth and development. Ca limitation symptoms have been described in various plants [30, 31]. In this study, we established that the seedlings of *L. chinensis*, the dominant forage grass in the eastern steppe of Eurasian continent, were tolerant to Ca limitation (Fig. 1A and B C). Two pieces of evidence excluded the possibility that the seedlings were not mature enough to take up nutrients from the growth environment. First, we observed that decreasing the [Ca^2+^]_ext_ in the growth medium resulted in a concomitant and significant decline in the Ca content in the leaves and roots of *L. chinensis* seedlings (Fig. 1D and E). Second, *L. chinensis* seedlings of the same age showed sensitivity to nitrogen limitation (Fig. S1). *L. chinensis* is a typical monocotyledonous perennial plant that can produce seeds and propagate through rhizome extension. In grasslands, *L. chinensis* usually takes three years to reach its full mature stage from seed germination. A long-term experiment is needed to fully understand the requirements of Ca for its growth and development.

Unique membrane properties may be responsible for conferring the Ca limitation tolerance phenotype of *L. chinensis* seedlings, which is supported by multiple lines of evidence in the study. Malondialdehyde (MDA) is a biomarker of oxidative damage of polyunsaturated fatty acids attached to the *sn*-2 position in glycerophospholipids within cell membranes [32]. Importantly, MDA content decreased in *L. chinensis* roots (Fig. 2C) while it increased in *Arabidopsis* roots in response to Ca limitation (Fig. 2E). Under Ca limitation condition, the superoxide dismutase (SOD) activity is increased and the anti-oxidative ability is enhanced. SOD carries out its anti-axidative duty by catalyzing hydroxy free radicals into H_2_O_2_. Therefore the roots have higher content of H_2_O_2_ and SOD activity under the Ca limitation condition than the CK. With decreasing amount of hydroxy free radicals, the extent of the membrane lipid peroxidation decreases, therefore MDA as the by-product of the membrane lipid peroxidation would decreases too. Ca limitation did not change CAT and POD activity in *L. chinensis* leaves and roots, indicating that no excess H_2_O_2_ was decomposed and that the Ca limitation-induced H_2_O_2_ accumulation did not cause oxidative stress.

Ca limitation significantly induced nonselective fluid-phase-based endocytosis in treated *Arabidopsis* roots, which led to a dramatic decrease in membrane selectivity [13]. By contrast, the *L. chinensis* root membrane maintained high selectivity even under the Ca limitation condition (Fig. 4). In addition, in both *Arabidopsis* and *L. chinensis*, PC, PE, PI, and PA were the four dominant glycerolipids. Ca limitation induced a significant increase in PC, PE, and PI content but a decrease in PA content in *Arabidopsis* roots [13], with no effects on any of the four glycerolipids in *L. chinensis* (Fig. 5A).

The relative abundance of each lipid type was significantly different between *Arabidopsis* and *L. chinensis* roots (Fig. 6), especially in terms of the PC to PE mole ratio (Fig. 6D). PC is an amphotropic lipid molecule with a hydrophilic head and a hydrophobic tail and plays a dominant role in stabilizing bilayer membranes. The PC to PE ratio is well maintained in animal cells, and an abnormally high or low ratio is recognized as a biomarker of disease progression [33]. In plants, the increased PC to PE ratio is a self-protective action that maintains the stability of the cell membrane under stress conditions [17]. The PC to PE ratio in *L. chinensis* was five times higher than that in *Arabidopsis* (Fig. 6D), suggesting that the membrane stability of *L. chinensis* is much higher than that of *Arabidopsis*.

FFAs are the only type of lipid that significantly rose in response to Ca limitation in *L. chinensis* roots (Fig. 5A). FFAs with 16 and 18 carbons participate in immune system regulation and response in plants, such as saturated FFA16:0 and FFA18:0, which help soybean (*Glycine max*) resist high temperature stress [34, 35]. The double bonds on the fatty acyl chain of cell membrane can reduce the packing density of adjacent lipids; moreover, reducing the number of double bonds in plant cells is helpful in maintaining cell membrane integrity and stability [36]. The significantly higher FFA16:0 and FFA18:0 content may therefore be beneficial for *L. chinensis* to maintain its root cell membrane stability.

We detected 31 unique lipid species in *L. chinensis* roots (Table S3). There were 12 unique PC species in *L. chinensis*, 11 of which contained odd fatty acid chains (Table S3). Pentadecanoic acid (C15:0) and heptadecanoic acid (C17:0) are odd fatty acid chains that accumulate to high levels in animals, plants, and microorganisms, being related to some diseases [37–39]. The specific functions of these PC molecules in *L. chinensis* roots need further investigation. Comparing these lipidomic data with those of other plants, we can draw both similarities and differences. For example, the unique PC36:0 in *Arabidopsis* roots and the unique PC35:2 and PC35:1 in *L. chinensis* are present in the epithelial cells of the dicotyledon common ice plant (*Mesembryanthemum crystallinum*) [40]. Unique LPC16:1 and LPE18:0 in *Arabidopsis* roots were previously detected in the roots of the monocot plant barley (*Hordeum vulgare*), but not the unique LPC14:0 in *L. chinensis* roots [41]. The unique lipid PC35:2 in *L. chinensis* roots was also previously reported in wheat (*Triticum aestivum*) leaves [42].

## Conclusion

*Arabidopsis* and *L. chinensis* are two plants belonging to different families. In addition, the *Arabidopsis* accession used here is an annual plant, while *L. chinensis* is a perennial species. They show significant differences in their growth sensitivity to Ca limitation and in their lipid composition. Further studies need to explore if similar differences exist in other plants. *L. chinensis* has highly stable membrane due to its unique membrane lipid composition, and our results suggest that this composition enables it to successfully adapt to a wide range of environmental conditions in the eastern Eurasian steppe.

## Methods

### Plant material and growth conditions

*L. chinensis* seeds were collected from wild *L. chinensis* plants with mature spikes in the Xilingol grassland and formally identified by Prof. Zhi Qi, the director of the *L. chinensis* propagation innovation team of the Inner Mongolia Autonomous Region. Voucher specimen of this material was deposited with identification number “LC ND 01” into the Grassland Plant Seeds Bank at the Grassland Health Center, Inner Mongolia University, These seeds were stored at 37 °C in an incubator to keep them dry. They were soaked in tap water at 4 °C for 5 d. Sunken seeds with higher germination rate were collected and surface-sterilized with 20% (w/v) NaClO and 0.1% (v/v) Triton X-100 for 2 h. This was followed by several washes with sterile water. The surface-sterilized seeds were placed on culture medium and kept in the dark at 30 °C for 2 d before being released in the growth chamber for 5–7 d [13].

*Arabidopsis* wild-type seeds from the Columbia-0 (Col-0) accession were surface-sterilized with 75% (v/v) ethanol for 15 min and then washed with 100% alcohol. The surface-sterilized seeds were sown on culture medium, stratified at 4 °C for 3 d in the dark, and then incubated in the growth chamber for 7 d. The culture medium contains 5 mM KNO_3_, 1 mM H_3_PO_4_, 1 mM MgSO_4_, 1 mM CaCl_2_, micronutrient and Fe concentrations equal to those in half-strength Murashige and Skoog medium, 5 mM MES, 1% (w/v) sucrose, 1% (w/v) agarose, pH 5.7 adjusted with Bis Tris Propane (Sigma).

The growth chamber was maintained at a light intensity of 75–100 µmol m^–2^ s^–1^; the light cycle was 12 h light/12 h dark, and the temperature range was 22 ± 2 °C, under which conditions both *L. chinensis* and *Arabidopsis* can achieve optimal growth [13].

### Ca content analysis of *L. chinensis*

After growth on medium with different concentrations of Ca^2+^ for 7 d, whole *L. chinensis* seedlings were washed with 1 mM CuSO_4_ for 1 min and with sterilized water three times to remove any residual mineral elements sticking to the root surface. The leaves and roots were separately collected and dried for 48 h at 80 °C. Weighted samples were grounded into fine powder with a grinder and digested with 5% (w/v) HNO_3_ for 48 h at 37 °C. The samples were centrifuged at 12,000 rpm for 10 min, and the supernatant was used for Ca content measurement by inductively coupled plasma optical emission spectrometry (ICP-OES, PQ 9000) [13].

### Endocytosis examination with confocal microscopy

*L. chinensis* seedlings were vertically grown on medium containing either no (0 mM) or 1 mM Ca^2+^ for 7 d. Seedlings with roots of about 2 cm in length were selected for confocal microscopy examination (Zeiss LSM710) of endocytosis activity as detailed previously [13].

### Extraction and measurement of *L. chinensis* root lipids

*L. chinensis* roots were sampled as described previously [13]. Then, samples were kept on dry ice and sent to the Lipidology Platform of the Institute of Genetics and Developmental Biology at the Chinese Academy of Sciences in Beijing for lipid profiling analysis using standard- and reverse-phase liquid chromatography–mass spectrometry (LC–MS), as detailed previously [43, 44]. Briefly, the tissues were extracted for lipids at 4 °C for 1 h with 200 µL ice-cold chloroform:methanol (1:1). These individual lipid species from polar lipids, including PC, PE, PS, PA, PI and LPC, LPE, LPS, were separated by a Phenomenex Luna 3 µ-silica column (internal diameter 150 × 2.0 mm) installed in an normal phase HPLC system (Agilent 1200) and further detected by a coupled triple quadrupole/ion trap mass spectrometer (AB SCIEX Exion UPLC-QTRAP 6500 PLUS). Phospholipids, sphingolipids, CL and glycerol lipids were separated by a Phenomenex Kinetex 2.6 μm C18 column (internal diameter 100 × 4.6 mm) and analyzed using a modified version of reverse phase (RP)-HPLC/ESI/MS/MS. Contents of all lipid species were quantified using LC-multiple reaction monitoring (MRM) in a combined workflow by referencing to corresponding internal standards [43, 44]. There were five biological *L. chinensis* root replicates for each treatment.

### Determination of MDA content, SOD, CAT, POD activity

The leaves and roots of *L. chinensis* and *Arabidopsis* seedlings were frozen and ground into powder for measuring MDA content with thiobarbituric acid (TBA), SOD activity with the nitro-blue tetrazolium (NBT) method, total CAT activity was assayed at 240 nm by measuring the rate of decomposition of H_2_O_2_ and total POD activity was measured at 470 nm by monitoring the oxidation of 3,30-dimethoxybenzidine according to the kit instructions (Comin, Suzhou).

### Localization of H_2_O_2_ production in plant tissues

For localizing H_2_O_2_ production, the leaves and roots of *L. chinensis* seedlings were stained with freshly prepared 1 mg/mL DAB solution and vacuum-infiltrated for 1 h in the dark. Afterward, the tissues were cleared in ethanol and stored in 20% (v/v) glycerol for photographing under a dissection microscope.

### Statistical analysis

The number of independent experiments and replicates per experiment are indicated in the table headings and figure legends. Data were plotted as means ± standard error of the mean. Statistical comparisons made include the Student’s t-test (2 groups), the ordinary One-way ANOVA followed by a Tukey multiple comparisons test (for more than 2 groups), and two-way ANOVAs with Tukey post hoc multiple comparison test (for 2 variables). We have also revised the figure legends.

### Electronic supplementary material

Below is the link to the electronic supplementary material.

Supplementary Material 1

Supplementary Material 2

## Data Availability

All data generated during this study are included in this published article and its supplementary information files, and the raw data used or analyzed during the current study are available from the corresponding author on reasonable request.

## Abbreviations

FFA: Free fatty acid
PC: Phosphatidylcholine
LPC: Lyso-PC
PE: Phosphatidylethanolamine
LPE: Lyso-PE
PI: Phosphatidylinositol
PA: Phosphatidic acid
PG: Phosphatidylglycerol
PS: Phosphatidylserine
MGDG: Monogalactosyldiacylglycerol
DGDG: Digalactosyldiacylglycerol
DAG: Diacylglycerols
TAG: Triacylglycerols
CL: Cardiolipin
FW: Fresh weight
DW: Dry weight
Tyr A23: Tyrphostin 23
Tyr A51: Tyrphostin 51
FM4-64: N-(3-triethylammoniumpropyl)-4-(6-(4-(diethylamino)phenyl)hexatrienyl)pyridinium dibromide)
LC–MS: Liquid chromatography–mass spectrometry
MDA: Malondialdehyde
SOD: Superoxide dismutase
DAB: 3,3′-diaminobenzidine
TBA: Thiobarbituric acid
NBT: Nitro-blue tetrazolium
