# Supplementary material for: Lipid profiling reveals Leymus Chinensis root insensitivity to Ca limitation
Source: BMC Plant Biol. 2023 Nov 30;23:602. doi: 10.1186/s12870-023-04627-8 (PMC10687902; doi:10.1186/s12870-023-04627-8)
Supplement: Supplementary file 2 — Supplementary Material 2 [file 12870_2023_4627_MOESM2_ESM.doc]

# *Supplementary Information*


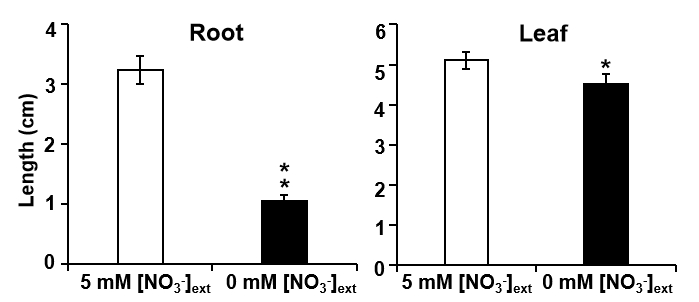


**Fig. S1: Effects of nitrogen deprivation on the *L. chinensis* growth.** Plant growth condition were same as the Fig. 1. Statistical analysis of root length and leaf length of *L. chinensis* seedlings under 5 (CK) or 0 mM [NO_3_^-^]_ext_ conditions. n = 20. *, *p* < 0.05 with student t-test.


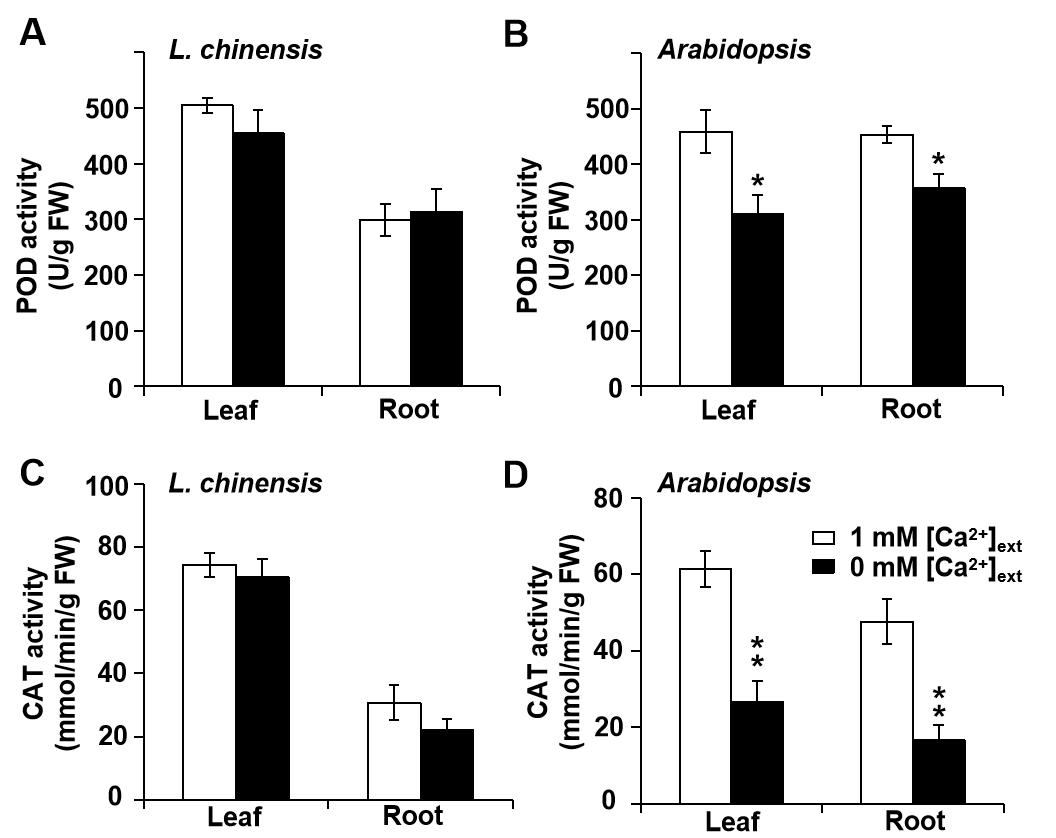


**Fig. S2:** **Detection of POD and CAT activity.** Plant materials were grown as in Fig. 1. **(A)** POD activity and **(C)** CAT activity in *L. chinensis* leaves and roots. **(B)** POD activity and **(D)** CAT activity in *Arabidopsis* leaves and roots. *n* = 5. **p* <0.05, ***p* < 0.01 with Student’s *t*-test.

**Table S1:** **Individual lipids species showing no significant changes to the Ca^2+^ deprivation in the *L. chinensis* roots.**

**Table S2: Individual lipids species showing significant changes to the Ca^2+^ deprivation in the *L. chinensis* roots. “**Ave” represents the average content of the lipid molecular species; SE represents the standard of five biological replicate; *p* represents the value with student t-test.

| **No.** | **Lipid species** | **1 mM [Ca**^2+^]**_ext_**  (nmol/g) | **0 mM [Ca**^2+^]**_ext_**  (nmol/g) | ***P*** | **0 mM [Ca**^2+^]**_ext_** /  1 mM [Ca^2+^]_ext_ |
| --- | --- | --- | --- | --- | --- |
|
| 1 | FFA16:0 | 64.4323 ± 3.1159 | 74.7729 ± 3.1818 | 0.0488 | 1.16 |
| 2 | FFA18:0 | 43.8401 ± 1.9441 | 53.5147 ± 2.8951 | 0.0241 | 1.22 |
| 3 | PC32:2 | 0.542 ± 0.0652 | 0.2551 ± 0.0406 | 0.0057 | 0.47 |
| 4 | PC32:1 | 0.1927 ± 0.0171 | 0.1246 ± 0.0221 | 0.0407 | 0.65 |
| 5 | PC43:2 | 0.0255 ± 0.0024 | 0.0403 ± 0.006 | 0.0498 | 1.58 |
| 6 | PC44:3 | 0.0132 ± 0.0021 | 0.0227 ± 0.0032 | 0.0372 | 1.73 |
| 7 | PA32:2 | 0.1783 ± 0.0189 | 0.0896 ± 0.0086 | 0.0027 | 0.5 |
| 8 | PA32:1 | 0.0456 ± 0.0073 | 0.0252 ± 0.0029 | 0.032 | 0.55 |
| 9 | PI33:3 | 0.1311 ± 0.0138 | 0.0769 ± 0.0176 | 0.0412 | 0.59 |
| 10 | PI32:2 | 0.062 ± 0.0053 | 0.0302 ± 0.0054 | 0.0029 | 0.49 |
| 11 | PE36:1 | 1.0679 ± 0.0512 | 1.2662 ± 0.0262 | 0.0088 | 1.19 |
| 12 | PE32:2 | 0.0982 ± 0.0064 | 0.0443 ± 0.0061 | 0.0003 | 0.45 |
| 13 | PE38:3 | 0.0785 ± 0.0037 | 0.0947 ± 0.0032 | 0.0106 | 1.21 |
| 14 | PE33:3 | 0.072 ± 0.006 | 0.0505 ± 0.0054 | 0.0286 | 0.7 |
| 15 | PE32:3 | 0.0254 ± 0.0022 | 0.0122 ± 0.0012 | 0.0007 | 0.48 |
| 16 | PE33:1 | 0.0249 ± 0.0032 | 0.0151 ± 0.0021 | 0.0327 | 0.61 |
| 17 | PE44:1 | 0.0184 ± 0.0019 | 0.0251 ± 0.0017 | 0.0334 | 1.36 |
| 18 | PE32:1 | 0.0157 ± 0.0017 | 0.0098 ± 0.001 | 0.019 | 0.62 |
| 19 | PE40:1 | 0.0138 ± 0.0014 | 0.0201 ± 0.0021 | 0.038 | 1.45 |
| 20 | PE35:5 | 0.0099 ± 0.001 | 0.006 ± 0.0011 | 0.0343 | 0.61 |
| 21 | MGDG32:2(18:2/14:0) | 0.0382 ± 0.0074 | 0.0121 ± 0.0019 | 0.0091 | 0.32 |
| 22 | DAG32:0(16:0/16:0) | 0.3135 ± 0.0219 | 0.1694 ± 0.0142 | 0.0006 | 0.54 |
| 23 | DAG34:0(16:0/18:0) | 0.2835 ± 0.012 | 0.1922 ± 0.027 | 0.0149 | 0.68 |
| 24 | DAG34:2(20:2/14:0) | 0.1952 ± 0.0202 | 0.2758 ± 0.0239 | 0.0328 | 1.41 |
| 25 | DAG34:1(16:0/18:1) | 0.1883 ± 0.0223 | 0.1288 ± 0.0109 | 0.0436 | 0.68 |
| 26 | DAG33:2(15:0/18:2) | 0.0946 ± 0.0098 | 0.0495 ± 0.0059 | 0.0044 | 0.52 |
| 27 | DAG32:2(18:2/14:0) | 0.071 ± 0.0072 | 0.0495 ± 0.0052 | 0.0414 | 0.7 |
| 28 | PG36:0 | 1.8118 ± 0.0869 | 2.1633 ± 0.0604 | 0.0105 | 1.19 |
| 29 | PG33:2 | 0.077 ± 0.007 | 0.0472 ± 0.0083 | 0.0251 | 0.61 |
| 30 | PG33:1 | 0.0093 ± 0.0005 | 0.0057 ± 0.0012 | 0.0243 | 0.62 |
| 31 | TAG50:2(16:0) | 0.2395 ± 0.0266 | 0.1006 ± 0.013 | 0.0015 | 0.36 |
| 32 | TAG50:2(18:2) | 0.1342 ± 0.02 | 0.0536 ± 0.0062 | 0.0049 | 0.37 |
| 33 | TAG48:0(16:0) | 0.0781 ± 0.0099 | 0.0286 ± 0.0018 | 0.0012 | 0.37 |
| 34 | TAG50:1(18:1) | 0.0581 ± 0.0068 | 0.0324 ± 0.0029 | 0.0085 | 0.56 |
| 35 | TAG49:2(18:2) | 0.0545 ± 0.0053 | 0.0132 ± 0.0018 | 0.0001 | 0.24 |
| 36 | TAG38:3(18:3) | 0.0222 ± 0.0015 | 0.0298 ± 0.0027 | 0.0399 | 1.35 |
| 37 | TAG40:4(18:3) | 0.0058 ± 0.0008 | 0.0095 ± 0.0004 | 0.0031 | 1.64 |
| 38 | TAG56:8(18:3) | 0.0029 ± 0.0003 | 0.0039 ± 0.0003 | 0.0324 | 1.36 |
| 39 | LPA16:0 | 0.3328 ± 0.0454 | 0.1577 ± 0.0104 | 0.0055 | 0.47 |
| 40 | LPC14:0 | 0.187 ± 0.0089 | 0.2186 ± 0.0068 | 0.0226 | 1.17 |
| 41 | LPS16:0 | 0.0104 ± 0.0018 | 0.0055 ± 0.0005 | 0.0272 | 0.52 |
| 42 | LPS18:1 | 0.0057 ± 0.0005 | 0.0081 ± 0.0005 | 0.0073 | 1.43 |
| 43 | LPS18:0 | 0.0047 ± 0.0001 | 0.0036 ± 0.0004 | 0.0467 | 0.78 |

**Table S3: The list of lipid molecular species existing in the *L. chinensis* but absent in the *Arabidopsis* roots.** Relative abundance of each lipid molecular species in its corresponding type lipid was calculated for each biological replicate. The average values of the five biological replicates were presented as “Ave (%)” with standard error (SE).

| **No.** | **Lipid species** | **Relative abundance of each lipid molecular species**  **in its corresponding type lipid (%)** |
| --- | --- | --- |
| 1 | PC35:2 | 0.412 ± 0.008 |
| 2 | PC41:4 | 0.236 ± 0.016 |
| 3 | PC35:1 | 0.187 ± 0.009 |
| 4 | PC39:4 | 0.133 ± 0.005 |
| 5 | PC41:5 | 0.122 ± 0.006 |
| 6 | PC39:3 | 0.114 ± 0.004 |
| 7 | PC41:3 | 0.078 ± 0.003 |
| 8 | PC43:2 | 0.051 ± 0.001 |
| 9 | PC43:3 | 0.043 ± 0.011 |
| 10 | PC41:6 | 0.022 ± 0.002 |
| 11 | PC44:1 | 0.022 ± 0.002 |
| 12 | PC43:1 | 0.015 ± 0.001 |
| 13 | PA40:6 | 0.21 ± 0.004 |
| 14 | PE41:5 | 0.505 ± 0.031 |
| 15 | PE39:3 | 0.313 ± 0.02 |
| 16 | PE39:4 | 0.077 ± 0.002 |
| 17 | MGDG35:6(18:1/17:5) | 0.683 ± 0.165 |
| 18 | MGDG37:5(19:3/18:2) | 0.148 ± 0.005 |
| 19 | DAG36:0(18:0/18:0) | 8.522 ± 1.044 |
| 20 | DAG34:0(16:0/18:0) | 4.027 ± 0.467 |
| 21 | DAG34:1(20:0/14:1) | 1.162 ± 0.234 |
| 22 | DAG34:1(21:0/13:1) | 1.038 ± 0.248 |
| 23 | DAG32:0(18:0/14:0) | 0.372 ± 0.068 |
| 24 | DAG34:1(17:1/17:0) | 0.17 ± 0.041 |
| 25 | DGDG36:1(16:0/20:1) | 0.145 ± 0.031 |
| 26 | TAG48:0(16:0) | 2.325 ± 0.476 |
| 27 | TAG38:3(18:3) | 0.968 ± 0.077 |
| 28 | TAG40:4(18:3) | 0.164 ± 0.018 |
| 29 | TAG59:5(18:3) | 0.064 ± 0.009 |
| 30 | LPC14:0 | 42.028 ± 4.607 |
| 31 | LPC15:0 | 6.861 ± 0.926 |

**Table S4: The list of lipid molecular species existing in the *Arabidopsis* but absent in the *L.chinensis* roots.** Relative abundance of each lipid molecular species in its corresponding type lipid was calculated for each biological replicate. The average values of the five biological replicates were presented as “Ave (%)” with standard error (SE).

| **No.** | **Lipid species** | **Relative abundance of each lipid molecular species**  **in its corresponding type lipid (%)** | **No.** | **Lipid species** | **Relative abundance of each lipid molecular species**  **in its corresponding type lipid (%)** |
| --- | --- | --- | --- | --- | --- |
| 1 | DAG31:1(18:1/13:0) | 0.031 ± 0.007 | 34 | MGDG34:5(18:3/16:2) | 1.141 ± 0.102 |
| 2 | DAG32:1(18:1/14:0) | 0.141 ± 0.009 | 35 | MGDG34:6(15:1/19:5) | 0.043 ± 0.012 |
| 3 | DAG32:2(16:1/16:1) | 0.098 ± 0.009 | 36 | MGDG34:6(15:2/19:4) | 0.032 ± 0.013 |
| 4 | DAG34:3(18:1/16:2) | 0.186 ± 0.023 | 37 | MGDG34:6(17:1/17:5) | 0.031 ± 0.01 |
| 5 | DAG34:4(18:2/16:2) | 0.066 ± 0.007 | 38 | MGDG34:6(17:2/17:4) | 0.039 ± 0.009 |
| 6 | DAG34:4(18:3/16:1) | 0.392 ± 0.031 | 39 | MGDG34:6(18:3/16:3) | 3.762 ± 0.377 |
| 7 | DAG34:6(18:3/16:3) | 0.075 ± 0.003 | 40 | MGDG34:6(18:4/16:2) | 0.172 ± 0.015 |
| 8 | DAG36:5(18:4/18:1) | 0.337 ± 0.033 | 41 | MGDG34:6(20:3/14:3) | 0.033 ± 0.004 |
| 9 | DAG36:6(18:4/18:2) | 0.177 ± 0.008 | 42 | MGDG36:3(18:0/18:3) | 0.244 ± 0.022 |
| 10 | DGDG34:4(16:2/18:2) | 0.096 ± 0.019 | 43 | MGDG36:6(21:4/15:2) | 0.174 ± 0.034 |
| 11 | DGDG34:4(18:3/16:1) | 1.018 ± 0.077 | 44 | MGDG36:6(21:5/15:1) | 0.121 ± 0.014 |
| 12 | DGDG34:5(16:2/18:3) | 0.158 ± 0.023 | 45 | MGDG36:6(22:4/14:2) | 0.061 ± 0.008 |
| 13 | DGDG34:6(18:3/16:3) | 0.465 ± 0.067 | 46 | PC36:0 | 2.852 ± 0.255 |
| 14 | DGDG36:6(16:1/20:5) | 0.348 ± 0.042 | 47 | PE33:0 | 0.009 ± 0.002 |
| 15 | DGDG36:6(17:0/19:6) | 0.39 ± 0.04 | 48 | PG30:0 | 0.189 ± 0.015 |
| 16 | DGDG36:6(17:1/19:5) | 0.261 ± 0.023 | 49 | PG32:3 | 0.177 ± 0.014 |
| 17 | DGDG36:6(17:3/19:3) | 0.253 ± 0.016 | 50 | PG36:7 | 0.041 ± 0.018 |
| 18 | DGDG36:6(21:4/15:2) | 0.102 ± 0.018 | 51 | PI32:3 | 0.155 ± 0.005 |
| 19 | LPA16:1 | 1.345 ± 0.024 | 52 | PI33:0 | 0.011 ± 0.001 |
| 20 | LPA18:0 | 1.68 ± 0.16 | 53 | PI35:0 | 0.019 ± 0.002 |
| 21 | LPC16:1 | 1.053 ± 0.053 | 54 | PS 38:4 | 1.344 ± 0.07 |
| 22 | LPE17:0 | 0.273 ± 0.042 | 55 | PS 38:5 | 0.261 ± 0.013 |
| 23 | LPE18:0 | 1.313 ± 0.125 | 56 | PS 40:5 | 0.121 ± 0.02 |
| 24 | LPE19:0 | 2.937 ± 0.384 | 57 | PS 40:6 | 0.07 ± 0.031 |
| 25 | MGDG29:2(16:0/13:2) | 0.071 ± 0.007 | 58 | TAG39:4(18:3) | 0.113 ± 0.02 |
| 26 | MGDG29:5(16:2/13:3) | 0.035 ± 0.005 | 59 | TAG49:0(17:0) | 0.032 ± 0.006 |
| 27 | MGDG31:3(18:2/13:1) | 0.124 ± 0.016 | 60 | TAG49:1(17:0) | 0.034 ± 0.009 |
| 28 | MGDG31:4(18:2/13:2) | 0.154 ± 0.017 | 61 | TAG49:3(18:2) | 0.023 ± 0.005 |
| 29 | MGDG34:3(18:1/16:2) | 0.334 ± 0.065 | 62 | TAG50:3(18:1) | 0.091 ± 0.011 |
| 30 | MGDG34:4(18:1/16:3) | 0.18 ± 0.023 | 63 | TAG50:3(18:2) | 0.15 ± 0.016 |
| 31 | MGDG34:4(18:2/16:2) | 0.468 ± 0.067 | 64 | TAG52:8(18:2) | 0.036 ± 0.006 |
| 32 | MGDG34:4(18:3/16:1) | 1.184 ± 0.04 | 65 | TAG52:8(18:3) | 0.061 ± 0.009 |
| 33 | MGDG34:5(18:2/16:3) | 0.543 ± 0.069 | 66 | TAG52:9(18:3) | 0.028 ± 0.005 |
